# The American Medical Association

**Published:** 1885-06

**Authors:** 


### THE AMERICAN MEDICAL ASSOCIATION.

### THIRTY-SIXTH ANNUAL MEETING, HELD AT NEW ORLEANS APRIL 28TH, 29TH, 30TH AND MAY 1ST, 1885.

Reported for the ATLANTA MEDICAL AND SURGICAL JOURNAL.

#### FIRST DAY.

TUESDAY, April 28th.

The thirty-sixth annual session of the American Medical Association was called to order at 11 o'clock in Tulane Hall, in New Orleans, by Dr. Samuel Logan, chairman of the Committee of Arrangements.

Dr. B. M. Palmer, of the Presbyterian Church, delivered an eloquent prayer, after which Dr. Henry F. Campbell, of Augusta, Ga., the President-elect, was introduced and took the chair.

Dr. Logan, the chairman of the Committee of Arrangements, then delivered an eloquent address of welcome on behalf of the profession and citizens of New Orleans.

The former Presidents and Vice-Presidents were invited to seats upon the platform.

Dr. Henry F. Campbell, the President, then delivered the annual address.

He alluded to great and meritorious physicians who formed this grand National Association; that his heart was gladdened as he saw the lengthening roll of distinguished membership. A new Pharaoh has arisen who hath not known the virtues of those who were departed, but their virtues would shine lustrously in all the future.

I see before me now the faces of a few of those who, over a generation ago, contributed in organizing the American Medical Association. He continued to eulogize the membership in their respective classes, and advised the present membership to make haste to honor all these groups of these Nestors of the Association. He reached the climax of his eulogistic effort by a loudly applauded peroration of praise to Dr. Davis, who presided at the birth of the Association, fostered and encouraged its development throughout its childhood, youth and maturity, until to-day he sits with us on this platform to enjoy its prosperity.

He next paid a tribute to men who were bravely engaged in the late war, and those who were kind benefactors of the South during the visiting epidemics, and congratulated the Association on its meeting in the hospitable city of New Orleans. He remarked: Warm and grateful will be our remembrance of the kind reception of the citizens of this Queen as she sits in the Crescent of the banks of the Mississippi river.

The American Medical Association, at each annual session, resembles the Congress of the nation, representing the varied interests of its broad domain. Each has a basis of representation, one in its legal character, and the other entrusted with the great scientific questions which embrace the life problems of the people. To remedy the defects in organization, or perfect those schemes already projected, are the measures that will engage the attention of the great and learned members of the American Medical Association.

He next proceeded to describe the "Doctor in the Courts."

Then showed the evils of malpractice suits, and handled, with masterly eloquence, the relation of the medical man to jurisprudence. In conclusion he said: Permit me to recommend the appointment of a committee to whom shall be given the subject of forensic medicine and the relation of the medical man to the tribunal of law.

Dr. Brodie moved that a vote of thanks be tendered Doctor Campbell for his able address, which was unanimously adopted.

The report of special committee, Austin Flint, M. D., chairman, in regard to the death of Prof. S. D. Gross, M. D., LL. D., being called for, Dr. T. G. Richardson, owing to the absence of

Dr. Flint, read the report, which was referred to the Committee on Printing.

Dr. Billings, of the United States Army, in the absence of Dr. Flint, said: I have to present two reports—first, the passing of a bill by Congress providing a fire-proof building for the National Medical Library, which building will be at once commenced. It will be located near the National Museum, in Washington, D. C. The second matter is the action of the Association in relation to the holding of the International Congress in 1887. It was moved and seconded that discussion on the subject be made the special subject at 12 M. Wednesday.

The reading of a report concerning a monument to Dr. Rush was deferred to Friday morning.

Dr. Logan read the titles of several papers, which were referred to the chairman of the section on Surgery.

The following physicians were made members by invitation: Drs. A. B. Miles, Edward Jones, C. J. Bickham, of New Orleans; A. C. Forte, of Philadelphia; J. G. Guthraux, of Louisiana; James R. Scarborough, of Kentucky.

Dr. Kinloch, of South Carolina, submitted, by request of the South Carolina Medical Association, the following resolution, which was adopted at its meeting in 1884:

*Resolved*, That the delegates from this Association be requested to present to the American Medical Association, at its next session, the report of the Committee on the Discovery of Sulphuric Ether, made to the Association at its last session, and request that body to take such action on the subject of the report as they in their judgment may determine in view of the claims therein set forth.

On motion, the report was referred to the section on Practice of Medicine, with a request for a report.

---

SECOND DAY.

WEDNESDAY, April 29th.

The Association was called to order at 10 o'clock by the President. After prayer, a telegram was read from ex-President Baldwin, of Alabama, expressing regrets at being absent.

Dr. H. D. Didama, of Syracuse, N. Y., then delivered the address in Medicine.

The address in Obstetrics was read by Dr. R. S. Sutton, of Pittsburg, chairman of the section of Obstetrics and Diseases of Women.

Dr. W. C. Van Bibber, of Maryland, read a portion of a paper on the Construction of a Health City in Florida. During the reading of this paper, the order of the day was called for. It was the consideration of the report of the Committee on Organization of the International Medical Congress.

Dr. John V. Shoemaker, of Philadelphia, rose and protested against the committee's report being received, alleging that the Association's delegates had been wholly ignored by the Committee on Invitations at the Copenhagen meeting, but that the new-code men present had openly threatened that they would prevent the acceptance of the invitation unless they received full recognition; as a consequence, they had been recognized, as was shown by the fact that they had been given some of the prominent positions with reference to the Washington meeting.

Dr. F. E. Daniel, of Texas, offered a resolution that the Committee on Nominations be instructed to nominate a President and other officers for the approaching International Medical Congress.

Dr. Billings denied Dr. Shoemaker's statement absolutely. His position was embarrassing; he spoke, not for the committee, but for what the committee had done—they had made no bargain with the new-code men. No new-code member had been consulted by the committee. The resolutions had been drawn at Washington, with the view of arranging for a necessary meeting at which to complete the preparations for the Congress.

Dr. Quinby said it was not a matter of men, but a matter involving serious principles; the committee had usurped power and had gone beyond their authority from the Associatoin, assuming executive and legislative functions, nominating officers, and the like. He closed by asking that the matter be referred back to the American Medical Association.

Dr. Keller thought that ninety-nine out of a hundred of the members of the Association believed that the committee were the creatures of the Association, and that they had exceeded their authority. He offered, as a substitute for Dr. Daniel's resolution, a motion that the President of the Association should appoint a committee, consisting of thirty-eight members, representing the army, the navy, the marine hospital service, the District of Columbia, and the territories (to be nominated by State delegations); this committee to meet the original committee to review, alter and amend the latter's action as it might deem best.

Dr. Cole, of California, seconded Dr. Keller's substitute. He would not charge the committee with having deliberately done anything directly in conflict with their understanding of their duty, but he believed they had erred egregiously. He believed the new code had been established for the purpose of punishing the so-called misdemeanors of the Association.

Dr. King, of Missouri, thought that this Association could not question the acts of the committee. He hoped the new thirty-eight would kindly cut the throats of the new-code men.

Dr. Shoemaker repeated that his statement was true, and said that new-code men had been appointed.

Dr. Daniel would not accept Dr. Keller's substitute. Texas had been ignored in the formation of the committee. He thought it would be a fatal mistake to establish the precedent of clothing this committee with the appointing power.

Dr. Sanders, of Tennessee, made the point, that this meeting was trying to undo the work of the committee. He moved that the committee's work be endorsed, with the proviso that they leave out the new-code men.

Dr. Roberts, of Philadelphia, spoke in favor of Dr. Sanders' resolution.

Dr. Smart, of Michigan, said that the committee had substituted legislative action for its executive duties—perhaps, not willfully; it had unduly transacted some of the duties which should have been relegated to this body. He suggested that each State should make its own nominations.

Dr. Ghent, of Texas, wished to endorse that part of the committee which did not include new-code men.

The Secretary read Dr. Sanders' resolution. It was lost by a vote of eighty-eight to one hundred and twenty-nine.

Dr. Keller's substitute was then put, and carried by a vote cf one hundred and thirty-one to ninety-two.

---

### THIRD DAY.

THURSDAY, April 30th.

The Association was called to order at 10 o'clock a. m. by the President.

After prayer by Rev. W. H. Waters, D. D., Dr. W. W. Potter, of Buffalo, N. Y., called up an amendment to the By-laws, offered last year by Dr. Foster Pratt, of Michigan, empowering each section to elect its own officers. After considerable discussion, it was postponed until the next annual meeting.

Dr. U. S. Davis, of Illinois, from the Standing Committee on Meteorological Conditions and their Relations to Prevalence of Disease, made a report, which was accepted, and the committee continued.

Dr. Davis also made the following report in reference to certain points in the Code of Ethics, which was adopted:

"*Whereas*, Persistent misrepresentations have been and are still being made concerning certain provisions of the Code of Ethics of this Association, by which many in the community, and some even in the ranks of the profession, are led to believe that these provisions exclude persons from professional recognition simply because of differences of opinions of doctrines; therefore be it

"*Resolved*, That clause 1, Art. iv., in the National Code of Medical Ethics, is not to be interpreted as excluding from professional fellowship on the ground of differences in doctrine or belief those who in other respects are entitled to be members of the regular medieal profession. Neither is there any other article or clause of the said Code of Ethics that interferes with the exercise of the most perfect liberty of individual opinion and practice.

"*Resolved*, That a voluntary disconnection or withdrawal from the medical profession proper is constituted by indicating to the public a sectarian or exclusive system of practice, or by belonging to an association or party antagonistic to the general medical profession.

"*Resolved*, That there is no provision in the Code of Medical Ethics in any way inconsistent with the broadest dictates of humanity; and the article which relates to consultations cannot be correctly interpreted as interdicting under any circumstances the rendering of professional services whenever there is a pressing or immediate need of them—on the contrary, to promptly meet the emergencies occasioned by disease or accident, or to give a helping hand to the distressed without unnecessary delay, is a duty fully enjoined on every member of the profession, by both the letter and the spirit of the entire code; but no such emergencies or circumstances can make it proper to enter into professional consultation with those who have voluntarily disconnected themselves from the regular medical profession in the manner indicated by the preceding resolution."

N. S. Davis, A. Y. P. Garnett, H. F. Campbell, Austin Flint, J, B. Murdock, Committee. Unanimously adopted.

The address of the chairman of the section on Surgery and Anatomy was then read by Dr. Duncan Eve, of Nashville, Tenn.

Dr. J. M. Toner read the report of the Committee on Publication. This report showed the *Journal* free from debt. The total income for the second year was $21,000; total expense, except editor's salary, $12,000. The publication of the *Journal* will continue in Chicago, and Dr. Davis will continue to edit it..

The report of the Committee on Erecting a Statue of Benjamin Rush, in the city of Washington, was adopted, and the following committee appointed: Dr. A. L. Gihan, of Washington; Dr. M. H. Henry, of New York; Dr. S. C. Gordon, of Maine; Dr. R. A. Kinloch, of South Carolina, and Dr. J. H. Murphy, of Tennessee.

The following committee, on that part of the President's address relating to Forensic Medicine, was announced: Drs. I. N. Quimby, New Jersey; C. Scott, Ohio; W. W. Dawson, Ohio; F.

E. Daniel, Texas; J. V. Shoemaker, Pennsylvania; Eugene Foster and H. F. Campbell, Georgia.

Dr. R. A. Kinloch, chairman of Committee on Nominations, read the following report, which was adopted:

For President, Wm. Brodie, of Michigan; for First Vice-President, Samuel Logan, of Louisiana; for Second Vice-President, J. Y. P. Garnett, of the District of Columbia; for Third Vice-President, Charles Alexander, of Wisconsin; for Fourth Vice-President, G. W. Peck, of Iowa; for Permanent Secretary, W. B. Atkinson, of Pennsylvania; for Assistant Secretary and Treasurer, R. J. Dunglison; for Librarian, C. H. A. Kleinschmidt, of the District of Columbia.

For Officers of Sections: Practice of Medicine.—Chairman, J. T. Whitaker, of Ohio; Secretary, B. L. Coleman, of Kentucky. Obstetrics and Diseases of Women.—Chairman, S. C. Gordon, of Maine; Secretary, —— Paine, of Texas. Surgery and Anatomy.—Chairman, N. Senn, of Wisconsin; Secretary, H. M. Mudd, of Missouri. Ophthalmology, Otology and Laryngology.—Chairman, Eugene Smith, of Michigan; Secretary, J. Fulton, of Minnesota. Diseases of Children.—Chairman, W. D. H. Ager, of Tennessee; Secretary, W. B. Lawrence, of Arkansas. Oral ond Dental Surgery.—Chairman, J. Marshall, of Illinois; Secretary, A. E. Baldwin, of Illinois. State Medicine.—Chairman, J. H. Rauch, of Illinois; Secretary, F. E. Daniel, of Texas. Committee on Necrology.—J. M. Toner, of the District of Columbia. Judicial Council.—R. A. Kinloch, of South Carolina; D. D. Sanders, of Tennessee; T. G. Richardson, of Louisiana; G. A. Ketchum, of Alabama; George Beard, of West Virginia; J. M. Toner, of the District of Columbia, and A. M. Pollock, of Pennsylvania.

---

### FOURTH DAY.

FRIDAY, May 1st.

The Association was called to order by the President. After prayer, the Committee on Nominations finished their report as follows: For Trustees of the *Journal*, Drs. J. M. Toner, of Washington; J. H. Hollister, of Chicago; E. M. Moore, of Rochester. For chairman of Committee of Arrangements, Dr. LeGrande Atwood, of St. Louis; for Assistant Secretary, Dr. Wm. Glasgow, of St. Louis; to fill the vacancy in the Judicial Council, caused by resignation of Dr. Brodie, of Detroit, Dr. J. K. Bartlett, of Milwaukee.

By Dr. Reed, of Ohio:

*Resolved*, That a committee of three be appointed by the President, whose duty shall be to devise a system for awarding, to those who may desire, honors for papers at the next meeting, and that said committee shall report through the Association *Journal*.

Adopted.

The resolution of Dr. Roberts, of Philadelphia, adopted in the section on State Medicine, and referred to the Association, was taken from the table and adopted.

This resolution recommends that each State and Territory establish a Board of Medical Examiners, whose certificate shall constitute the only license to practice medicine.

Dr. White, of Richmond, Va., read the address of the chairman in Ophthalmology, Otology and Laryngology, which was referred to the Committee on Publication.

Dr. J. H. Pope, of Texas, chairman of the section in Diseases of Children, gave an abstract of his address. The address was referred to the Committee on Publication.

The address of the chairman of the section on Dental and Oral Surgery was ordered sent direct to the *Journal* without reading.

The proposed amendment to the By-laws, creating a section on Medical Jurisprudence, proposed by Dr. Quimby, was laid over to next year.

Dr. Toner, of Washington, made a report on Necrology.

The proposed amendment to the By-laws, dividing the section of Ophthalmology and creating a section on Laryngology, was indefinitely postponed.

The amendment proposed in reference to higher education was laid on the table.

Dr. Morris H. Henry, of New York, was elected a delegate to the British Medical Association.

After the usual resolutions of thanks, the Association adjourned to meet in St. Louis on the first Tuesday in May, 1886.

